# The role and therapeutic significance of the anoikis pathway in renal clear cell carcinoma

**DOI:** 10.3389/fonc.2022.1009984

**Published:** 2022-09-29

**Authors:** Jin Wang, Xiaochen Qi, Qifei Wang, Guangzhen Wu

**Affiliations:** Department of Urology, The First Affiliated Hospital of Dalian Medical University, Dalian, China

**Keywords:** KIRC, anoikis, bioinformatics analysis, cell death, target therapy

## Abstract

Anoikis is a specialized mode of programmed cell death. Specifically, once cells detach from the original extracellular matrix, an apoptotic program is initiated, preventing colonization of the cells in distant parts of the organ. Therefore, both distant metastasis and colonization of cancer cells rely on the anoikis resistance of cancer cells. Bioinformatics analysis was performed to confirm the relation of anoikis to kidney renal cell carcinoma (KIRC). To construct a prognostic model for patients with KIRC, we investigated several genes of the anoikis pathway most closely related to KIRC and also contrasted the effects of common anticancer drugs on the KIRC pathway. Besides KIRC, we explored the expression of anoikis-related genes in various other cancers. We classified patients with KIRC into three clusters based on the coefficients and mRNA expression levels of anoikis-related genes selected using the GSVA algorithm. We used the GDSC database to predict the response of the anoikis pathway to common anticancer drugs and explored the potential targets of the anoikis pathway in KIRC. We then analyzed the response of common immunotherapies to the anoikis pathway to analyze the correlation between anoikis and immune checkpoint inhibitor therapy. Finally, eleven cancer-related genes were screened and a prognostic model was constructed using LASSO regression.

## Introduction

Anoikis is a programmed cell death that is initiated immediately after cell-extracellular matrix (ECM) interactions are disrupted upon cells detaching from the original ECM. ECM receptors of the integrin family are essential to inhibit anoikis. Integrin receptors not only provide the physical links to the cytoskeleton, but they are also responsible for signal transduction between cells and the ECM, which is necessary for cell migration, proliferation and survival (1–5). Anoikis ensures tissue and developmental homeostasis and prevents the growth and attachment of non-adherent cells to inappropriate substrates to avoid the colonization of distant organs. Therefore, suspension cells that do not undergo anoikis can proliferate at different ectopic sites of ECM proteins. The phenomenon of anoikis resistance in cancer cells is becoming known as the ability of cancer cells to further evolve their mechanism of distant metastasis (6–9). In previous studies, anoikis resistance has been reported in a variety of cancers. For example, in gastric and lung cancers, cancer cells induce anoikis resistance and metastasis through epidermal growth factor receptor (EGFR) signaling (10, 11). In melanoma, we observed that increased N-cadherin expression promotes anoikis resistance (12). In a variety of cancers, development of anoikis resistance has been associated with metastasis; however, such reports are rare in KIRC.

Anoikis is regulated by two pathways, one of which is intrinsic mitochondrial perturbation and the other is triggered by extrinsic cell surface death receptors. The intrinsic pathway mainly involves the transfer of the pro-apoptotic proteins Bax and Bak from the cytoplasm to the outer mitochondrial membrane (OMM), which then forms channels within the OMM. This increases mitochondrial permeability and releases cytochrome C, which leads to the formation of the “apoptosome” (13). The extrinsic pathway is primarily initiated by ligand binding of members of the tumor necrosis factor receptor (TNFR) superfamily, resulting in the formation of the death-inducing signaling complex (DISC) (14). Both pathways lead to the activation of caspases and downstream molecular pathways, which ultimately activate DNA endonucleases and induce apoptosis.

Renal cell carcinoma (RCC) is one of the most common malignant tumors of the urinary system. According to GLOBOCAN statistics in 2020, RCC has a high incidence and mortality with an increasing trend (15). Due to the strong compensatory capacity of the kidney, RCC is clinically insidious, resulting in the early detection of kidney injury. Kidney renal cell carcinoma (KIRC), comprising approximately 85% of RCC cases, is the most typical RCC. Hemorrhage, necrosis, cystic degeneration, and calcification of the renal parenchyma are frequently observed in KIRC patients. The clinical treatment of KIRC has attracted increasing attention because of the poor prognosis and high mortality rate of patients with KIRC.

In this study, 34 anoikis-related genes (including SRC, AKT1, and BCL2) were selected to represent the expression of the anoikis pathway using the GSEA database. We used bioinformatics analysis to explore the correlation between the expression levels of anoikis-related genes and the progression of KIRC development and clinicopathological features with the aim to accurately interpret the role of anoikis mechanisms in KIRC. Finally, LASSO regression was used to screen for anoikis-related genes to establish a KIRC prognostic model. These results can serve as a guide for clinical diagnosis, treatment, and prognosis.

## Results

### Widespread mutations in anoikis-related genes

**Figure 1:**
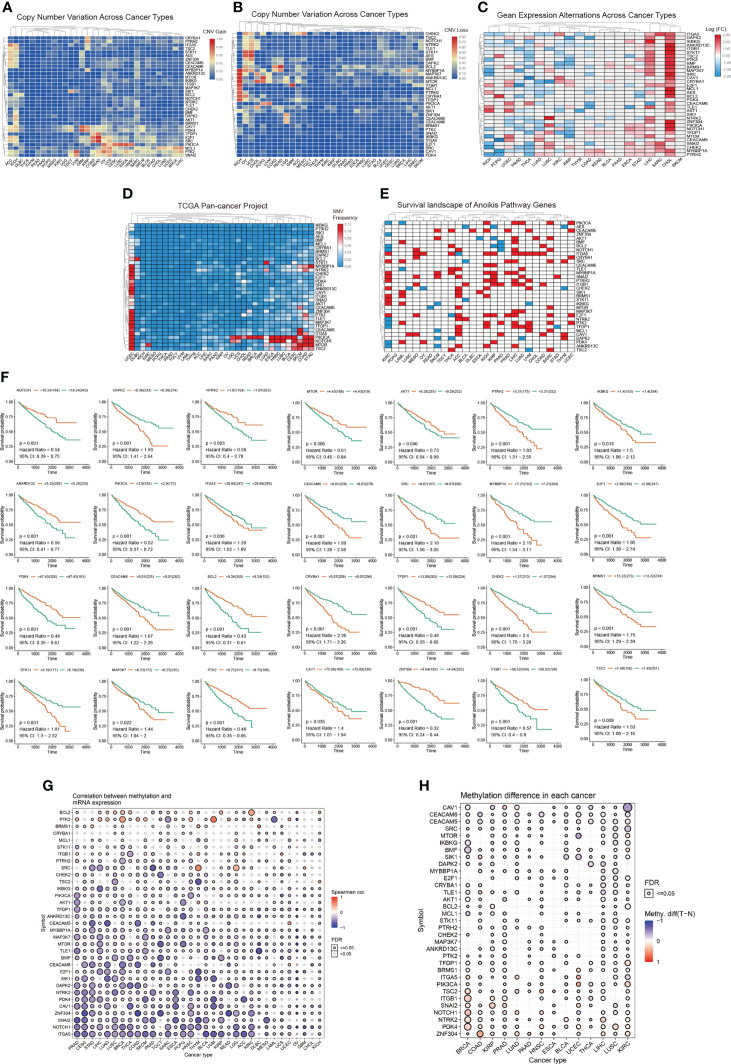
**(A, B)** CNV frequencies of the 34 anoikis pathway genes in the 32 tumor types. Red and blue colors indicate a CNV gain and loss, respectively. **(C)** Expression levels of anoikis-related genes in 20 cancers. The color code bars show the corresponding values of log2(FC) on the right, with the shift from red to blue corresponding to values ranging from 3.00 to -3.00. **(D)** SNV frequencies of the 34 anoikis pathway genes in the 32 tumor types. Red and blue colors indicate a high and low frequency, respectively. **(E)** The three categories of anoikis-related genes: risk genes (Red), protective genes (Blue), and no statistically significant genes (White, p > 0.05). **(F)** Survival curve analysis of all statistically significant genes in KIRC in TCGA samples. Red and blue colors represent high and low expression groups, respectively. **(G, H)** Different degrees of methylation in each cancer and correlation between methylation and mRNA expression levels. The color depth of the ring on the right indicates the comparison between the P-value and 0.05. The color bar indicates the degree of difference and the correlation coefficient.

To investigate the expression changes and mutations of anoikis-related genes in multiple tumors, we used sample data from the TCGA database. We determined the mRNA expression (**Figure 1C**) as well as the frequency of CNV (**Figures 1A, B**), and SNV (**Figure 1D**) in anoikis-related genes in various tumors. In the panorama of mRNA expression, we observed changes in expression levels of anoikis-related genes in a variety of cancer types. Specifically, NOTCH1, CRYBA1, CAV1, and ITGA5 were highly expressed in KIRC, whereas MYBBP1A, TFDP1, PIK3CA, ATK1, TLE1, BRMS1, BMP, TSC2, STK11, and ITGB1 were expressed at low levels in KIRC. The Perl and R languages were used to analyze the CNV and SNV data obtained from the TCGA database. The results showed that CAV1, PDK4, TFDP1, E2F1, SRC, PIK3CA, MCL1, PTK2, and SNAI2 presented CNV gains in different cancer types. Simultaneously, BCL2, MYBBP1A, MAP3K7, ANKRD13C, MTOR, and CHEK2 showed CNV loss. From the SNV results, we can conclude that anoikis-related genes have single nucleotide variations in 32 tumors to varying degrees. The survival landscape and correlation analysis of gene expression (**Figure 1E**) revealed that anoikis-related genes were most strongly correlated with KIRC. As shown in the survival curve (**Figure 1F**), most patients with increased anoikis-related gene expression tended to have a poor prognosis. The methylation data of the anoikis gene set obtained from the GSCAlite platform in pan-cancer showed that the anoikis gene set had a strong correlation with methylation in a variety of cancers, and the methylation differences in a variety of cancers were high, and the results were statistically different (**Figures 1G, H**).

### Cluster analysis for three sample groups

**Figure 2:**
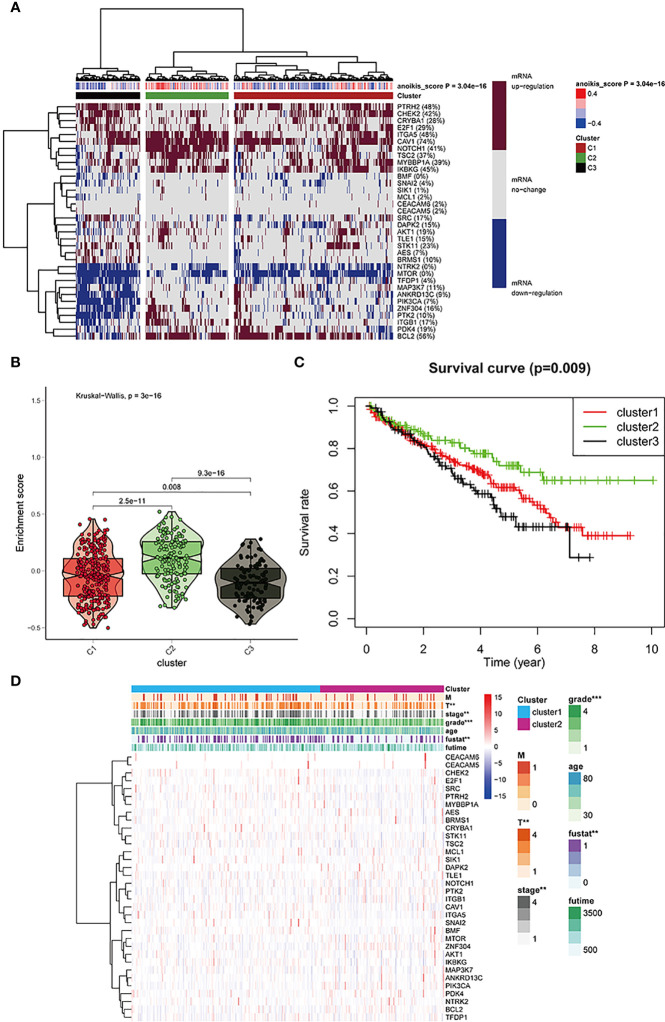
**(A)** All KIRC samples are divided into three groups according to different levels of anoikis score: high/medium/low expression group (cluster1, 2, and 3). The dark red/blue represent mRNA expression increased/decreased, respectively. The redder/bluer the color, the closer the anoikis score is to 0.4/-0.4, respectively. Three groups divided through cluster analysis: red/olive green/black represent clusters 1,2, and 3, respectively. **(B)** The enrichment scores of the three clusters. The color differentiation was the same as **Figure 2A**. **(C)** Survival curves are based on three clusters. **(D)** The correlation between anoikis scores and the clinicopathological characteristics of KIRC patients. *p < 0:05, **p < 0:01, and ***p < 0:001.

Based on mRNA expression, cluster analysis was used to classify all samples obtained from TCGA into three clusters. The anoikis scores indicated different mRNA expression levels (**Figure 2A**). The obtained clusters of C1, C2, and C3 represented anoikis scores as active, normal, and inactive, respectively. The violin plot (**Figure 2B**) shows the degree of difference of the three clusters based on anoikis score numerical difference. P value less than 0.05 indicates that the clusters obtained by cluster analysis have statistically significant inter-group differences, which can be used for subsequent analysis. The survival curve (**Figure 2C**) re-emphasizes the difference in prognosis among the three clusters, which indicates that the difference analysis results obtained later are related to prognosis. Both the violin plot and survival curve emphasize the characteristics of these three sample groups: the group with no change in anoikis pathway had a good prognosis, whereas the group exhibiting anoikis pathway downregulation had poor prognosis.

The anoikis pathway genes in the three clusters were found to be closely associated with cancer grading and survival outcome in clinicopathology, as indicated by the heatmap in **Figure 2D**.

### Expression of histone-modified genes and classical oncogenes in three clusters

**Figure 3:**
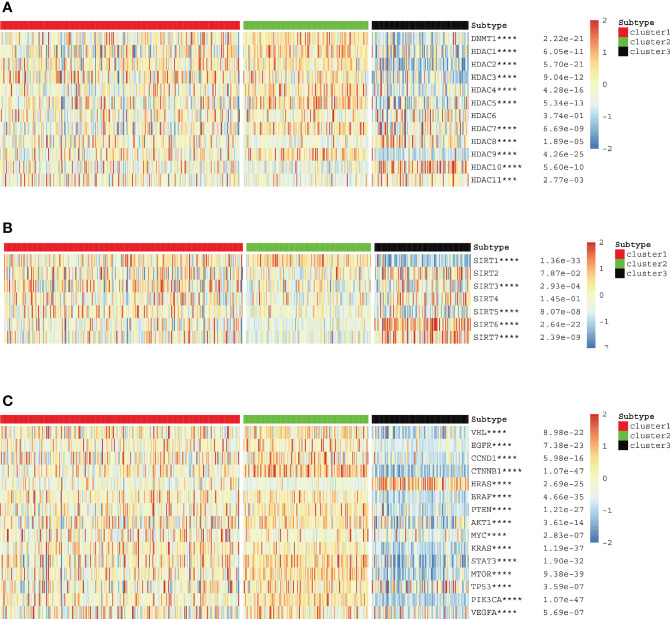
**(A–C)** HDAC, sirtuin and potentially targetable classical family genes the correlation with anoikis scores respectively.

We used cluster analysis to obtain three clusters and investigated the correlation between classical oncogenes, HDAC family genes, sirtuin family genes, and KIRC. Based on the generated heat maps and p-values, we found that all classical oncogenes were closely associated with tumor progression. (∗p<0.05, **p<0.01, ***p<0.001, ∗∗∗∗p < 0.0001) (**Figure 3C**). The results showed that most sirtuin and HDAC family genes, in addition to SIRT2, SIRT4, and HDAC6, were also strongly associated with tumorigenesis and progression (**Figures 3A, B**).

### Correlation analysis between drug therapy and anoikis scores

**Figure 4:**
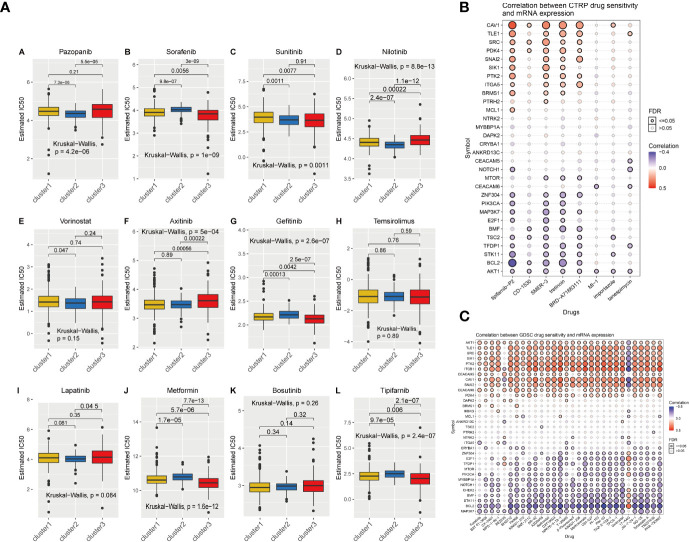
**(A)** The IC50 prediction of KIRC cells treated with a common tumor-targeted drug. C1, C2, and C3 indicate Cluster1, Cluster2, and Cluster3. **(B, C)** The correlation between the sensitivity of drugs results in various cancer obtained from the CTRP or GDSC database and the mRNA expression levels of anoikis-related genes.

To further explore the potential value of anoikis in the clinical treatment of patients with KIRC, we identified potential drugs targeting the anoikis pathway based on drug sensitivity predictions from the GDSC database. The specific prediction results of drug sensitivity of the 12 clinically common targeted drugs are shown in the box chart (**Figure 4A**). The effect of most drugs is different from the changes in anoikis expression. This score can provide a more precise guide for the development of future drug treatments for cancer. We also collected data on potential drug-mRNA expression correlations revealed by the CTRP and GDSC databases. The results showed that most anoikis-related genes have potential responsiveness to targeted drugs or small molecule drugs (**Figures 4B, C**).

### Immune cell infiltration based on ssGSEA

**Figure 5:**
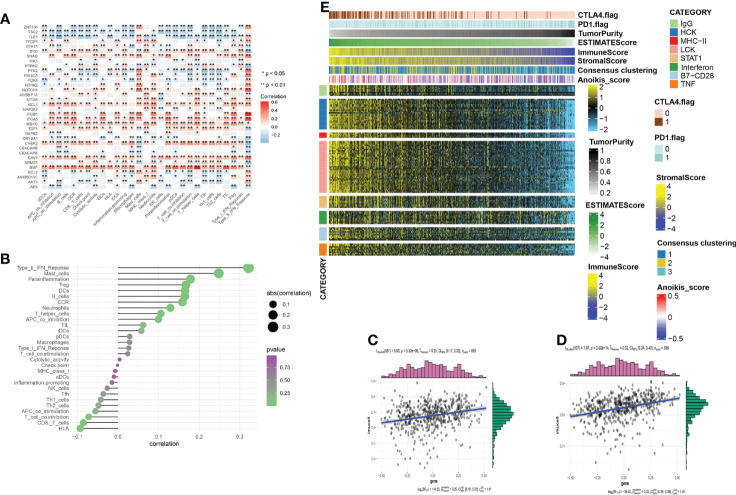
**(A)** The correlation between the immune infiltration and the anoikis-related genes. Red/blue represent a positive/negative correlation, respectively. (*p < 0:05, **p < 0:01). **(B)** The plot shows the degree of correlation. The area of the circle represents the abs (correlation) and the color bar on the right side shows the p value. **(C, D)** Mast cell and Type_II_IFN_reponse were selected, and the scatter diagram shows the correlation with anoikis score. **(E)** To identify the meta-genes of major expression vectors associated with immunotherapy, we screened the samples for some genes associated with 8 inflammatory factors. The expression of these genes and other immunotherapy-related scores in the KIRC samples.

Immunotherapy has attracted much attention for cancer treatment. To examine the feasibility of anoikis-related immunotherapy in KIRC, we investigated the relationship between immunity and anoikis. Immune cell infiltration was closely associated with the anoikis pathway genes, as shown in the heatmap in **Figure 5A**). The bubble plot shows the sequence of the correlation between immune infiltration-related cells and anoikis (**Figure 5B**). The two immune cells most strongly correlated with anoikis were selected to analyze their relevance and the results showed a positive correlation (**Figures 5C, D**). Metastatic patients can be cured by therapeutically blocking the CTLA-4 and PD-1 point receptors on T cells (16). Therefore, immune checkpoint blocking therapy is widely used in patients (17). We hypothesize that the small sample size is the main reason for this phenomenon. We used heatmaps to show the differences and trends in metagene expression of different immune markers among different samples and sorted the samples according to ESTIMATEScore and TumorPurity (**Figure 5E**). The results showed that with the increase of tumor purity / the decrease of immune infiltrating cells or stromal cells, the level of anoikis-score showed an downward trend, and the expression level of metagene identifying various immune inflammatory factors showed a downward trend.

### Establishing a prediction model using LASSO regression

**Figure 6:**
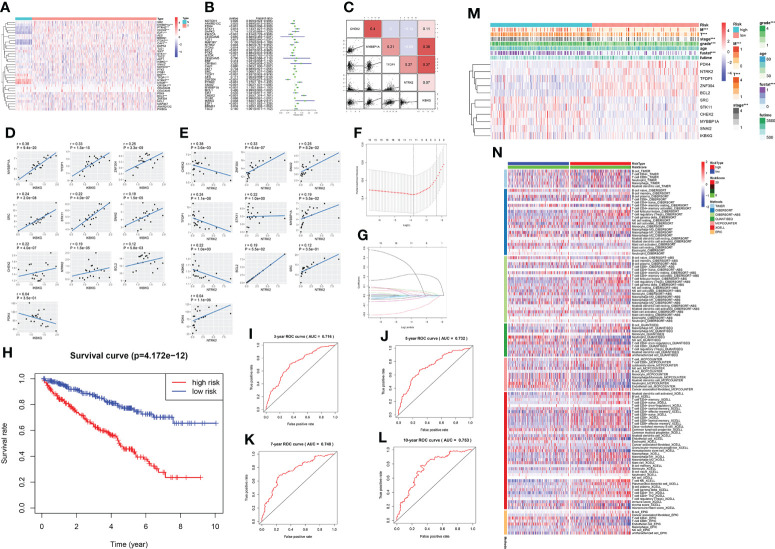
A prediction model of KIRC using anoikis-related genes. **(A)** Expression of anoikis-related genes in patients with KIRC. Red and blue colors represent upregulation and downregulation, respectively. N (green) represents the normal sample; T (red) represents the tumor sample (*p < 0:05, **p < 0:01, and ***p < 0:001). **(B)** Hazard ratio analysis with 95% confidence intervals and p values for the anoikis-related genes. **(C)** Co-expression relationship between 5 anoikis-related genes with significant statistical significance. Their regression relationship is displayed with scatter plots. The correlation coefficient is represented by a color: a positive correlation is red; a negative correlation is blue; the deeper the color, the greater the correlation. **(D)** The scatter diagram shows the correlation of IKBKG and other genes, including MYBBP1A, TFDP1, ZNF304, SRC, STK11, SNAI2, CHEK2, BCL2, PDK4, and NTRK2. **(E)** The scatter diagram shows the correlation of NTRK2 and other genes, including CHEK2, ZNF304, SNAI2, TFDP1, STK11, MYBBP1A, IKBKG, BCL2, IKBKG, SRC, and PDK4. **(F, G)** The LASSO coefficient profiles of anoikis-related genes in KIRC. Eleven genes were selected using the LASSO Cox regression analysis. **(H)** The survival curve obtained using this model. Red and blue colors correspond to the high- and the low-risk groups, respectively. **(I–L)** ROC curve represents data of 3, 5, 7, and 10 years. AUC is marked at the head (3 years: 0.716; 5 years: 0.732; 7 years: 0.748; and 10 years: 0.753). **(M)** Heatmap shows the correlation of the eleven selected genes and their clinicopathological characteristics in the two groups. The color bar represents the expression of genes; red and blue represent upregulation and downregulation, respectively. **(N)** Heatmap for immune responses based on different algorithms among the high and low-risk groups. Different algorithms are represented by different colored area bars.

By analyzing the data obtained from TCGA database for the control and KIRC groups, we found that 18 of the 34 anoikis pathway genes were significantly different between the two groups (**Figure 6A**). The forest diagram shows the results of hazard ratio analysis. The genes NOTCH1, ANKRD13C, PDK4, PIK3CA, NTRK2, BCL2, PTK2, TFDP1, ZNF304, and BRMS1 conferred a protective effect, whereas STK11, ITGA5, SRC, PTRH2, SNAI2, MYBBP1A, CHEK2, IKBKG, and E2F1 exerted a risk effect (p <0.05 was considered significant) (**Figure 6B**). Five randomly selected genes with statistically significant effects on KIRC (P < 0.05) were selected for co-expression analysis (**Figure 6C**); two of which, IKBKG and NTRK2, showed strong co-expression relationships with other genes (**Figures 6D, E**). Specifically, MYBBP1A, TFDP1, ZNF304, SRC, STK11, SNAI2, CHEK2, BCL2, PDK4, and NTRK2 positively correlated with IKBKG expression, while CHEK2, ZNF304, SNAI2, TFDP1, STK11, MYBBP1A, IKBKG, BCL2, SRC, and PDK4 were positively correlated with NTRK2 expression. Appropriate genes were selected to construct prediction models using LASSO regression analysis (**Figures 6F–G**). The receiver Operating Characteristic curve (**Figure 6H**) demonstrated the predictive prognostic performance of the new KIRC patient survival model. Survival curves (**Figures 6I–L**) for four different time nodes (3, 5, 7, and 10 years) were obtained. The results showed that the AUC values of all observed time nodes were greater than 0.7 (0.7, considered predictive). We then reclassified the cancer samples into high-risk and low-risk groups based on the optimal cut-off calculated by the survival package. We screened their survival curves and 11 genetic features using LASSO regression (**Figure 6M**). After obtaining the LASSO regression model, we demonstrated the correlation between the risk score and immune cells in various immune infiltration algorithms; according to the sequence of the risk score based on the sample risk score (**Figure 6N**).

### Predictive model analysis and validation

**Figure 7:**
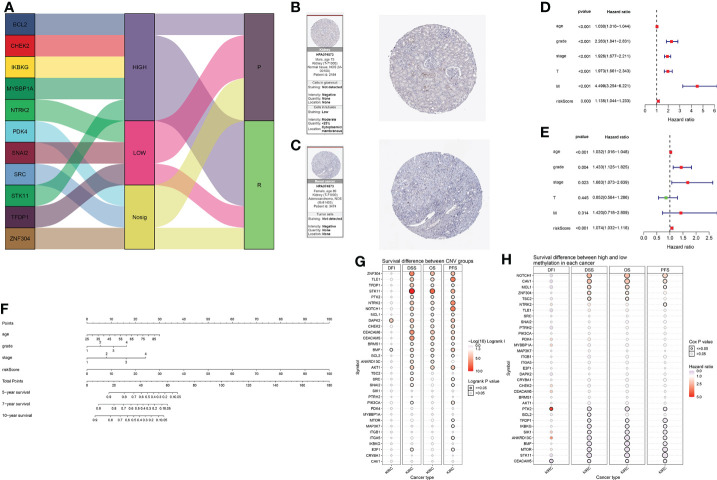
**(A)** Sankey diagrams used to summarize the data on the eleven selected genes, including their expression and prognosis. **(B, C)** The association between anoikis-related genes and KIRC was confirmed at the protein level. Immunohistochemical images of protein NTRK2 was obtained from the HPA website. **(D)** Forest plot of univariate Cox analysis. **(E)** Forest plot of multivariate Cox analysis. **(F)** Nomogram of the prediction model. The total score is calculated using ABC, from which 5-, 7-, and 10-year survival rates can be obtained. **(G)** Correlation between CNV mutations of different anoikis-related genes and survival coefficient in KIRC. **(H)** Correlation between methylation of different anoikis-related genes and survival coefficient in KIRC.

The expression and prognosis of the 11 selected genes are shown in the Sankey diagram (**Figure 7A**). In addition, we display immunohistochemistry data for the proteins of NRTK2 obtained from the Human Protein Atlas (HPA) website (**Figures 7B, C**). Univariate and multivariate Cox regression analyses were performed. All 11 genes were shown to be risk factors, except for T and M (**Figures 7D, E**). Finally, the prediction model and total points of age, grade, stage, and risk scores were derived using the survival data of KIRC patients at 5, 7, and 10 years; the results are shown in the nomogram (**Figure 7F**). The prognosis of renal cell carcinoma is closely related to patient age and postoperative pathological stage. Appropriate inclusion of these clinical data enhances the accuracy of the prognostic model. We collected survival, CNV and methylation data from the GSCAlite database to show the correlation between the survival landscape with CNV and methylation (**Figures 7G, H**).

## Discussion

Anoikis is a form of programmed cell death (PCD) and it was first described in 1994. Anoikis induces cells detached from the extracellular matrix to initiate apoptosis, thereby preventing colonization of cells to distant tissues (18). The ability of cancer cells to resist anoikis has attracted widespread attention because anchorage-independent growth and epithelial-mesenchymal transition are not only two characteristics of anoikis resistance but also crucial steps in the progression of cancer and metastatic colonization (9). Cancer cells can overcome anoikis through several mechanisms to ensure survival, which in turn promotes their invasion and metastasis. Anoikis resistance is caused by growth proteins, pH, transcriptional signaling pathways, and oxidative stress (19). Moreover, the tumor microenvironment is considered to contribute to anoikis resistance in cancer cells by enhancing oxidative stress, triggering epithelial-mesenchymal transition, and causing metabolic dysregulation in cancer cells. Anoikis resistance in cancer cells plays a pleiotropic role in several of these steps, making it a key point in anti-metastatic targeted therapies.

In this study, we analyzed the correlation between gene mutations and the expression of anoikis-related genes and pathways. To determine whether anoikis could be a potential target for KIRC treatment, we compared clinical information on KIRC samples to those on controls using the TCGA database. Moreover, we investigated the therapeutic effects of common targeted cancer treatment drugs on KIRC and selected genes related to anoikis and KIRC to establish cancer prognostic models.

KIRC samples were classified into three clusters based on the RNA expression levels of anoikis-related genes; these three clusters represented high, normal, and low expression of anoikis-related genes, respectively. Based on the three clusters, we constructed survival curves for the anoikis-related genes. We found that downregulation of the anoikis pathway had the lowest survival values, which is consistent with our previous conjecture that the anoikis pathway plays a protective role in KIRC. To confirm the role of some drugs in the treatment of KIRC, we performed GDSC analyses, which indicated that the majority of targeted drug treatments for KIRC were associated with effects on the expression levels of genes in the anoikis pathway. These analyses contribute to the clinical treatment of KIRC. These results provide new insights into the development of targeted drugs for the treatment of KIRC.

Among recent reports on cancer immunotherapy, acetylating histones and enhancing T-cell killing are widely accepted treatment options (20, 21). By analyzing the expression of some classical oncogenes (such as KRAS and VHL) and immune genes associated with histone acetylation in the three clusters, we found that most of these genes are associated with the anoikis pathway. PD-1 and CTLA-4 are two common T-cell immunotherapy targets that are commonly used in KIRC treatment (22). In contrast to the direct cytotoxicity of conventional chemotherapy, immune checkpoint blockade drives cytotoxic T cells in the body to target tumor cells, thereby activating the suppressed immunity within the tumor (23). After examining the responsiveness of the two anoikis score clusters to PD-1 and CTLA-4 inhibitor targets, we were surprised to find that anoikis-inactive clusters were more likely to respond to anti-CTLA-4 treatment. However, after the Bonferroni correction, the results were not statistically significant.

Subsequently, we used HR and co-expression analysis on 40 of these anoikis pathway genes to compare their expression in cancer to that in normal tissues. Finally, 11 genes in the anoikis pathway were screened using LASSO regression to construct a survival prediction model for patients with KIRC. The induction of BCL2 modifier expression can induce mitochondria-dependent apoptosis in cancer cells (24). This indicates that BCL2 is a proapoptotic factor, which is consistent with results from our prediction model. Similarly, upregulation of NTRK2 expression promotes cancer cell migration and invasion (25), which is also consistent with our predictions. We believe that this model will have great application in future clinical studies on cancer treatment and prognosis.

## Materials and methods

### Data acquisition and analysis

Based on the GSEA website (http://www.gsea-msigdb.org/gsea/index.jsp) (26), 34 genes closely associated with anoikis-related pathways were selected for further analysis. The TCGA database project provides genetic and clinical information on cancer (https://tcga-data.nci.nih.gov/tcga/) (27). The TCGA database is a comprehensive genome-wide gene expression collection created by large-scale gene sequencing and multidimensional analysis. It is used to classify and detect genetic changes caused by tumor development. We obtained genetic data by studying gene expression levels, CNV and SNV of genes, clinical survival landscapes, and clinicopathological features. The Perl language was used to analyze gene expression data, and TBtools was used to conduct visual analysis. Survival curves of all KIRC-related critical genes were plotted to visualize the role of these genes in KIRC progression. The statistical significance level was set at p < 0.05.

### Cluster analysis in three groups

Based on mRNA expression levels, we calculated the anoikis score to quantify the expression of genes involved in the anoikis pathway. Cluster analysis was performed to obtain the following three clustered samples based on the anoikis score: Cluster 1, Cluster 2, and Cluster 3, which indicated high, normal, and low expression of anoikis pathway genes, respectively. The three clusters were verified using violin plots and survival curves, and heatmaps were used to display their gene expression levels and clinicopathological characteristics. The statistical significance level was set at p < 0.05.

### Histone modification-associated genes and classical oncogenes

Histone modifications such as methylation and acetylation are directly associated with gene mutations. Sirtuin (SIRT) is a highly conserved deacetylase gene family (28). In addition to their roles in cancer initiation, promotion, and progression, SIRTs play an important role in many other biological processes. They exhibit oncogenic and tumor-suppressive properties owing to differences in their cellular environment and experimental conditions (29). Histone deacetylases (HDACs) are part of a large family of enzymes that play a key role in many biological processes, mainly through their inhibitory effects on transcription (30). Meanwhile, the study of HDAC in cancer pathogenesis has been widely reported, as it is significant for chromosome structure formation and regulation of gene expression (21). We identified classical and newly discovered oncogenes, such as AKT1 and mTOR. Using a heatmap, we visualized different expression patterns of canonical oncogenes in the three clusters. We also explored other genes relevant to histone modification, such as sirtuin and HDAC. The statistical significance level was set at p < 0.05.

### Exploration of cancer treatment

GDSC is a publicly accessible database that contains information about drugs, genes, and tumors (31). It provides free and publicly available genomic data for identifying potential cancer therapeutic targets. The GDSC database can also provide results of drug susceptibility testing of cancer cells for different targeted drugs. To estimate the IC50 of samples in the three clusters using ridge regression, we implemented the pRRophetic algorithm in R using the “pRRophetic” package (32). Using a boxplot and correlation chart, we visually compared the half-inhibitory concentration values of each drug within the three clusters. The statistical significance level was set at p < 0.05.

### Immune cell infiltration

We used ssGSEA analysis combined with the TCGA database to quantify immune cell infiltration levels; the results of their correlation are presented as a heatmap. In this study, we selected 29 immune cells and regulators from molecules involved in innate and adaptive immunity and described their correlation with anoikis-related genes using histograms. The two most strongly correlated immune cells were selected for further analysis. The open-source R packages “data. table”, “dplyr”,”Tidy”, “GGplot2”, and “ggStatsplot” were used for statistical analysis of data and plotting heatmaps and scattermaps. Pd-1 (22) and CTLA-4 (33) have been reported as regulatory receptors related to T-cell killing of tumor cells, which are closely related to immune checkpoint blocking cancer therapy. The correlation among CTLA-4, PD-1, and anoikis scores is shown in the correlation matrix. Cluster1 was abandoned for grouping the two classification studies because its expression was at the middle level.

### Construction of the prediction model

The heatmap shows the difference in the expression levels of anoikis-related genes between control and KIRC tissues and the co-expression relationship between any two anoikis-related genes. Univariate COX regression was used to analyze the relationship between anoikis pathway-related genes and KIRC patient risk indicators (stage, grade, etc.). The LASSO regression model was constructed with the “glmnet” package. Risk score =∑ Ni =1 (Expi∗Coei); N, Coei, and Expi represent the gene number, regression correlation coefficient, and gene expression level obtained by LASSO regression analysis, respectively. The “survival” package was used to calculate the cutoff values for risk scores in the tumor group. Based on this cutoff value, the samples were divided into high- and low-risk groups, and survival curves were drawn. Finally, the “Meier survival ROC” package was used to draw the ROC curve and obtain the AUC value. The correlation of clinicopathological features between the low- and high-risk groups is shown by heatmaps. The statistical significance level was set at p < 0.05.

### Prediction model establishment and verification

A Sankey diagram created with the “ggalluvial” package was used to visualize multiple attributes of risk and protective genes that were statistically significant in the HR analysis. We obtained protein information from the HPA website (www.proteinatlas.org/) (34). Univariate and multivariate Cox regression analyses were used to illustrate the correlation between age, stage, grade, T (tumor), M (metastasis), and risk score in the model. RStudio software was used for all statistical analyses. The nomogram was drawn by the “rms” package in R. Lastly, to consolidate our conclusions, we performed immunohistochemistry experiments using KIRC clinical specimens for NTRK2, a key molecule in this model. The statistical significance level was set at p < 0.05.

### R software packages

For survival analysis and COX regression analysis, we used the survival, limma, and survivor packages. For cluster analysis, we used the consensus cluster and hclust packages. For LASSO regression analysis, we used the net package. For plotting, we used the ggplot2, ggstatsplot, corrplot, vioplot, forest plot, and RMS packages.

## Data availability statement

The original contributions presented in the study are included in the article/**Supplementary Material**, further inquiries can be directed to the corresponding authors.

## Author contributions

GW and QW designed the research methods. JW, and XQ performed the experiments and analyzed the data. JW and XQ drafted and revised the manuscript. All authors approved the version of the manuscript to be released and agreed to be responsible for all aspects of the work. JW, and XQ contributed equally to this study. All authors contributed to the article and approved the submitted version.

## Acknowledgments

We thank The Cancer Genome Atlas (TCGA) project for providing us with the publicly available data. This project is supported by Dalian Young Stars of Science and Technology Fund (No. 2022RQ010), the Liaoning Province Doctoral Research Startup Fund Program (No. 2021-BS-209) and Natural Science Foundation of Liaoning Province of China (No. 2021-MS-278). We would like to thank Editage (www.editage.cn) for English language editing.

## Conflict of interest

The authors declare that the research was conducted in the absence of any commercial or financial relationships that could be construed as a potential conflict of interest.

## Publisher’s note

All claims expressed in this article are solely those of the authors and do not necessarily represent those of their affiliated organizations, or those of the publisher, the editors and the reviewers. Any product that may be evaluated in this article, or claim that may be made by its manufacturer, is not guaranteed or endorsed by the publisher.

## Glossary

**Table:**

KIRC	Kidney renal clear cell carcinoma
PD-1	Programmed cell death protein 1
CTLA-4	Cytotoxic T lymphocyte-associated protein 4
LASSO	Least absolute shrinkage and selection operator
ECM	extracellular matrix
RCC	renal cell carcinoma
GSEA	Gene Set Enrichment Analysis
TCGA	The Cancer Genome Atlas
CNV	The copy number variation
SNV	Single-nucleotide variation
SIRT	Sirtuins
HDAC	Zinc-dependent histone deacetylases
GDSC	Genomics of Drug Sensitivity in Cancer
MTOR	Mammalian target of rapamycin
TME	The tumor microenvironment
ROC	receiver operating characteristic
AUC	area under the curve
HPA	The Human Protein Atlas
TNM	tumor node metastasis
HR	hazard ratio
NTRK2	Neurotrophic receptor tyrosine kinase 2
EGFR	epidermal growth factor receptor
OMM	outer mitochondrial membrane
ACC	Adrenocortical carcinoma
BLCA	Bladder Urothelial Carcinoma
BRCA	Breast invasive carcinoma
CESC	Cervical squamous cell carcinoma and endocervical adenocarcinoma
CHOL	Cholangiocarcinoma
COAD	Colon adenocarcinoma
COAD/READ	Colon adenocarcinoma/Rectum adenocarcinoma Esophageal carcinoma
DLBC	Lymphoid Neoplasm Diffuse Large B-cell Lymphoma
ESCA	Esophageal carcinoma
GBM	Glioblastoma multiforme
GBML	Glioma
HNSC	Head and Neck squamous cell carcinoma
KICH	Kidney Chromophobe
KIRC	Kidney renal clear cell carcinoma
KIRP	Kidney renal papillary cell carcinoma
LAML	Acute Myeloid Leukemia
LGG	Brain Lower Grade Glioma
LIHC	Liver hepatocellular carcinoma
LUAD	Lung adenocarcinoma
LUSC	Lung squamous cell carcinoma
MESO	Mesothelioma
OV	Ovarian serous cystadenocarcinoma
PAAD	Pancreatic adenocarcinoma
PCPG	Pheochromocytoma and Paraganglioma
PRAD	Prostate adenocarcinoma
READ	Rectum adenocarcinoma
SARC	Sarcoma
SKCM	Skin Cutaneous Melanoma
STAD	Stomach adenocarcinoma
STES	Stomach and Esophageal carcinoma
TGCT	Testicular Germ Cell Tumors
THCA	Thyroid carcinoma
THYM	Thymoma
UCEC	Uterine Corpus Endometrial Carcinoma
UCS	Uterine Carcinosarcoma
UVM	Uveal Melanoma.
