# Supplementary material for: The role and therapeutic significance of the anoikis pathway in renal clear cell carcinoma
Source: Front Oncol. 2022 Sep 29;12:1009984. doi: 10.3389/fonc.2022.1009984 (PMC9557223; doi:10.3389/fonc.2022.1009984)
Supplement: Supplementary file 6 [file Presentation_1.pptx]

## Slide 1
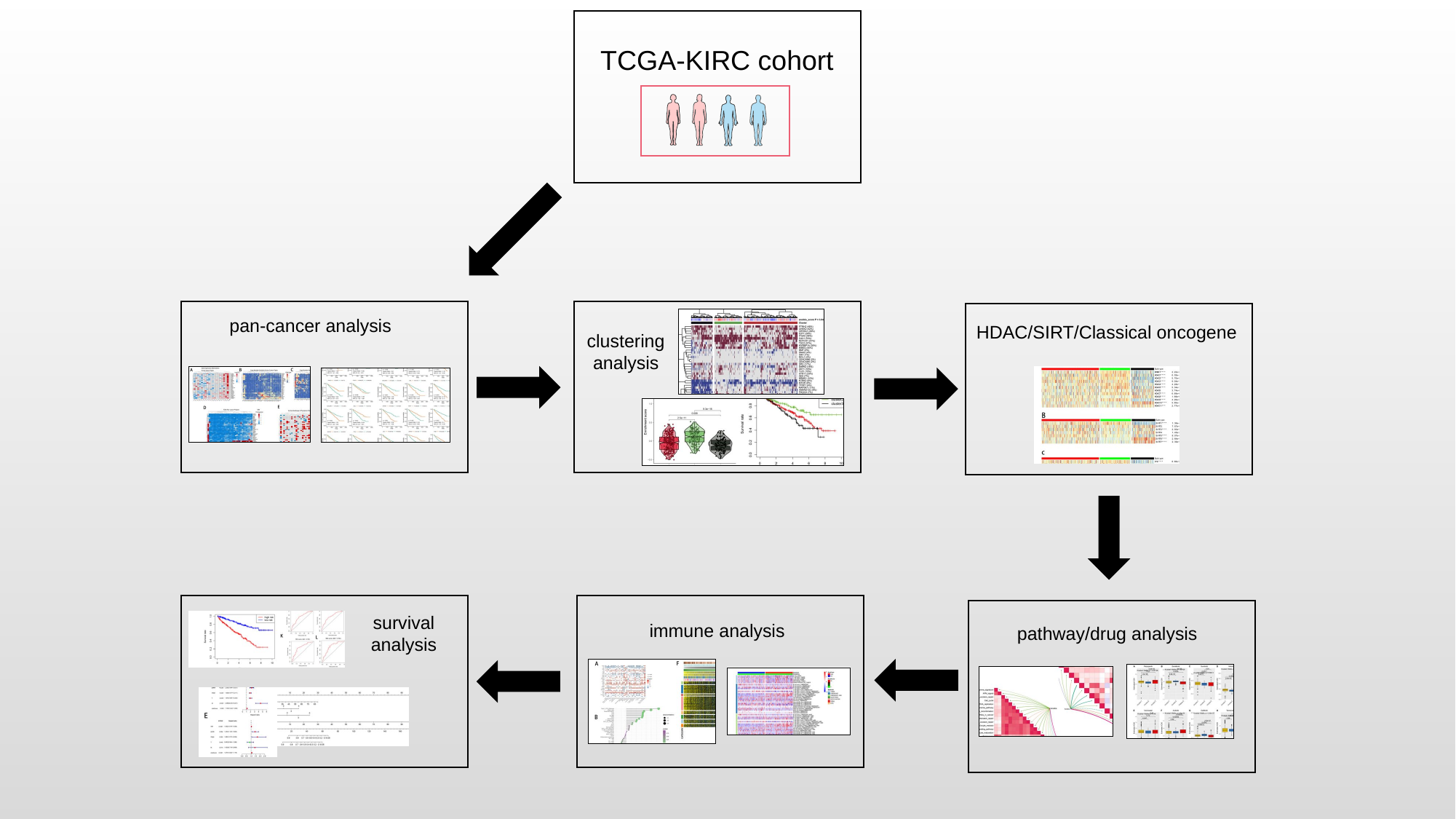

TCGA-KIRC cohort
pan-cancer analysis
HDAC/SIRT/Classical oncogene
clustering analysis
survival analysis
immune analysis
pathway/drug analysis
